# Gun violence in United States during the second year of the COVID-19 pandemic

**DOI:** 10.3389/fpubh.2023.950475

**Published:** 2023-03-06

**Authors:** Paddy Ssentongo, Anna Ssentongo, Emily S. Heilbrunn, Vernon M. Chinchilli

**Affiliations:** ^1^Department of Medicine, Penn State College of Medicine and Milton S. Hershey Medical Center, Hershey, PA, United States; ^2^Department of Public Health Sciences, Penn State College of Medicine and Milton S. Hershey Medical Center, Hershey, PA, United States

**Keywords:** gun violence, COVID-19 pandemic, public health crisis, United States, hotspots analyses

## Abstract

**Objective:**

In the first year of the COVID-19 pandemic, gun violence (GV) rates in the United States (US) rose by 30%. We estimate the relative risk of GV in the US in the second year compared to the first year of the pandemic, in time and space.

**Methods:**

Daily police reports of gun-related injuries and deaths in the 50 states and the District of Columbia from March 1, 2020, to February 28, 2022, were obtained from the GV Archive. Generalized linear mixed-effects models in the form of Poisson regression analyses were utilized to estimate state-specific rates of GV.

**Results:**

Nationally, GV rates during the second year of the pandemic (March 1, 2021, through February 28, 2022) remained the same as that of the first year (March 1, 2020, through February 28, 2021) (Intensity Ratio = 0.996; 95% CI 0.98, 1.01; *p* = 0.53). Nevertheless, hotspots of GV were identified. Nine (18%) states registered a significantly higher risk of GV during the second year of the pandemic compared to the same period in the first year. In 10 (20%) states, the risk of GV during the second year of the pandemic was significantly lower compared to the same period in the first year.

**Conclusion:**

GV risk in the US is heterogeneous. It continues to be a public health crisis, with 18% of the states demonstrating significantly higher GV rates during the second year of the COVID-19 pandemic compared to the same timeframe 1 year prior.

## Introduction

Understanding the spatial distribution and temporal evolution of gun violence (GV) in the second year of the COVID-19 pandemic is crucial to ascertain its burden and optimize public health interventions tailored to regions with the greatest need. In the United States (US), gun violence increased by 30% in the first year of the pandemic, and the risk was higher in over half of the states (1). A recent US study concluded that large-scale racial disparities exist in exposure to neighborhood firearm violence, which grew during the pandemic (2). Additionally, it was postulated that controlling the pandemic through mass vaccination could lead to a concomitant reduction in GV rates. The present analysis estimates spatiotemporal dynamics of GV in the second year compared to the first year of the pandemic.

## Methods

Individual events data were acquired from The Gun Violence Archive (GVA), an independent not-for-profit organization that compiles comprehensive and accurate information about GV in the US (3) https://www.gunviolencearchive.org. The data points of interest were information about daily events, the location of the incident (street address, city, and state), and the number of individuals killed or injured. State population data and other demographic characteristics (age, sex, and race) were extracted from the US Census Bureau, Department of Commerce database (4). We compared the rate of GV between the first year of the pandemic (defined as March 1, 2020, through February 28, 2021) to the second year of the pandemic (March 1, 2021, through February 28, 2022), using the first year as the reference period.

To estimate the intensity ratio (IR) of GV comparing the second year to the first year of the pandemic, we applied a generalized linear mixed-effects model in the form of Poisson regression analysis with a logarithm link function for each state. For the overall US analysis, we applied a generalized linear mixed-effects model in the form of a Poisson regression analysis as described above with three additional features: a first-order autoregressive process to account for the correlation across the time intervals; random effect for the state; four covariates based on census data (each state's median age, Black-White ratio, Hispanic-White ratio, and male-female ratio).

Estimates of spatial distributions of GV during the second year of the pandemic vs. the first year was performed using spatial relative risk surfaces (5). Statistical significance level was set at *p* < 0.01 for spatial relative risk surface and *p* < 0.05 for all other analyses. All analyses were performed with the R statistical language (R Development Core Team 2020 Version 3.0.6) and SAS Version 9.4.

## Results

### Risk of gun violence during the second year of the COVID-19 pandemic

**Figure 1:**
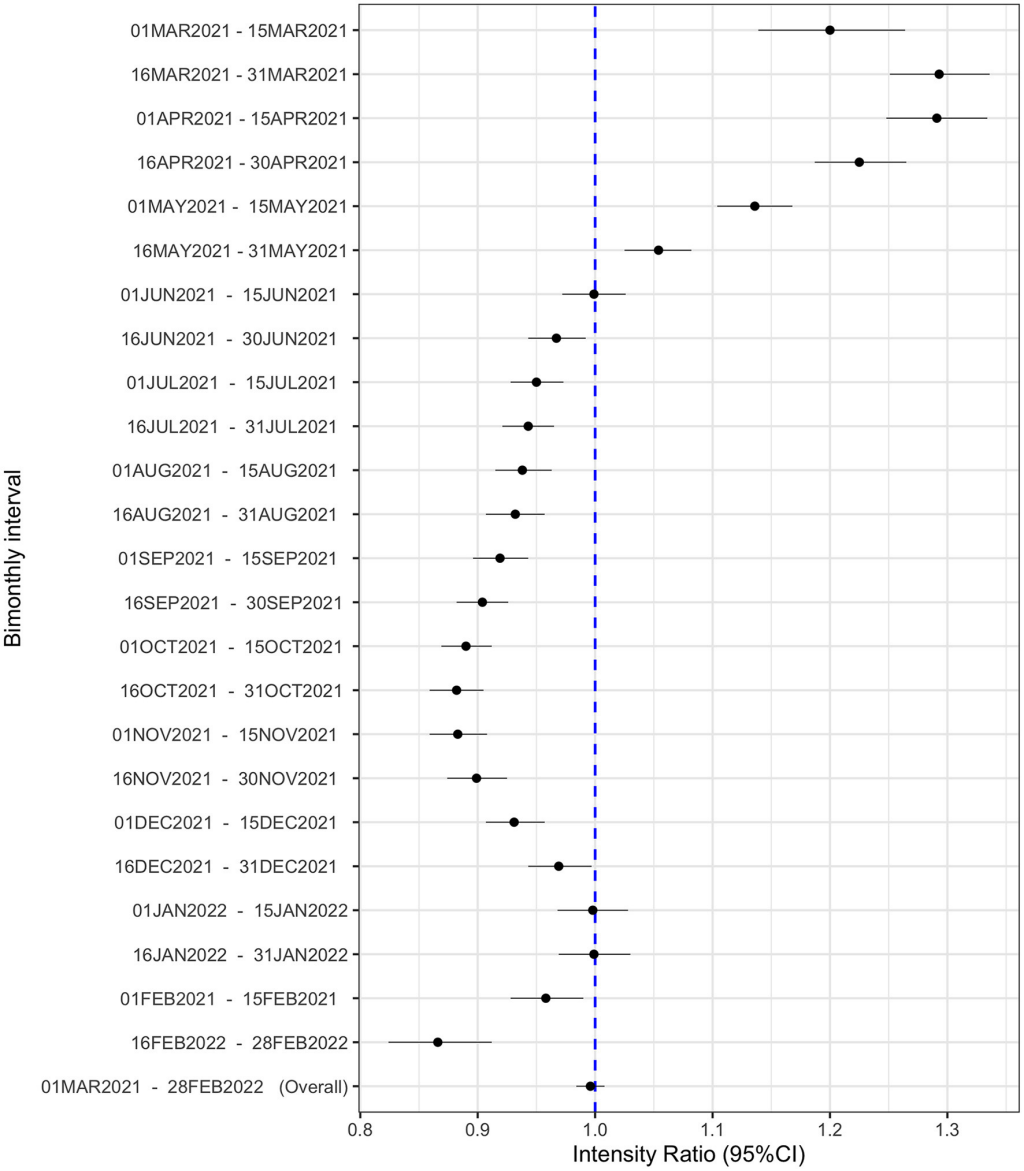
Bimonthly intervals for GV intensity during the second year compared to the first year of the pandemic. Bimonthly interval-specific intensity ratio (IR) and their 95% confidence intervals of GV. The dashed blue line in the forest plots represents the null estimate. IR greater than one indicates higher intensity of GV during the first compared to the second year of the COVID-19 pandemic.

**Figure 2:**
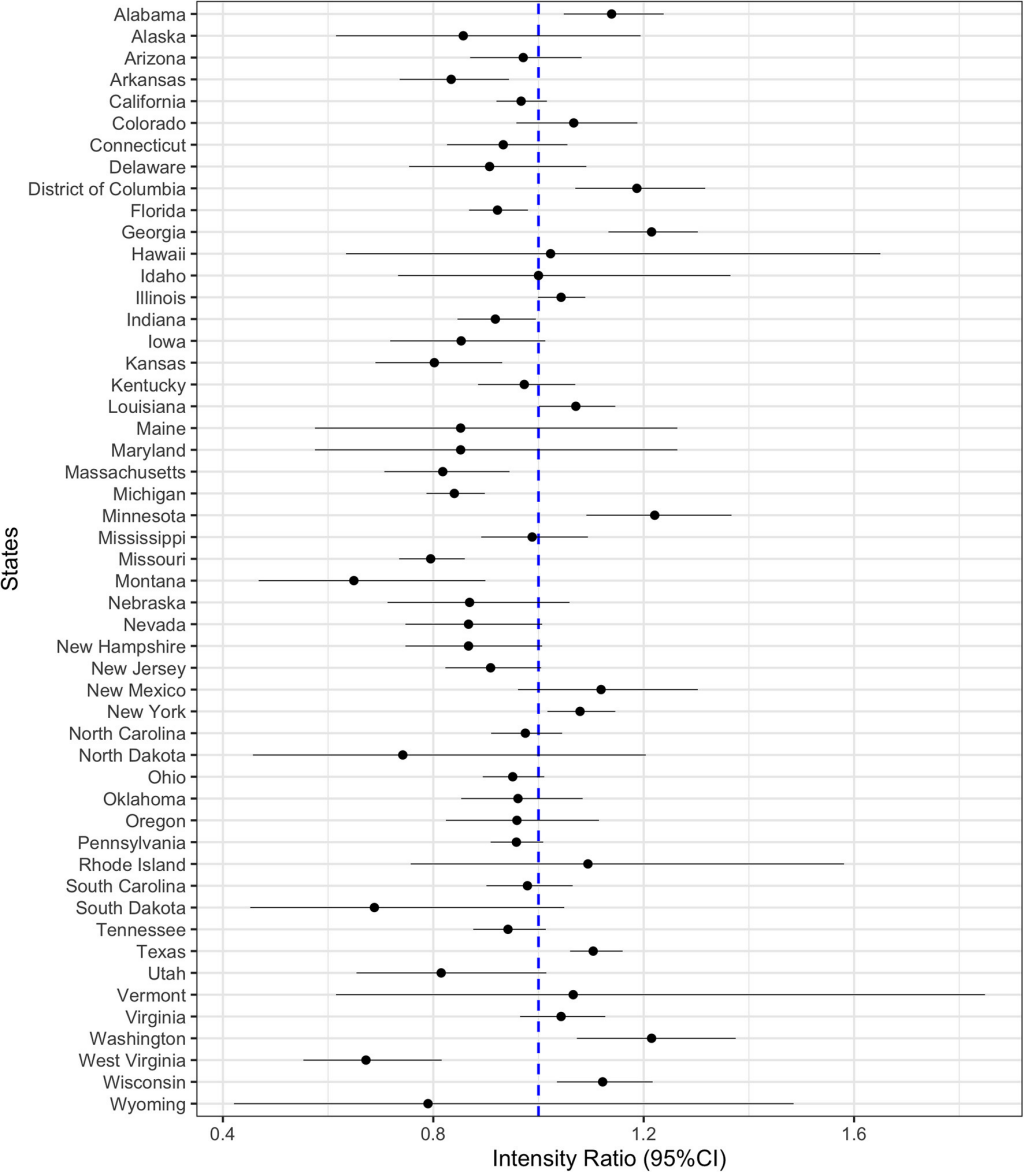
State-specific intensity of GV during the second year compared to the first year of the pandemic. State-specific intensity ratio (IR) and their 95% confidence intervals of GV. The dashed blue line in the forest plots represents the null estimate. IR greater than one indicates higher intensity of GV during the second year than in the first year of the pandemic.

On a national level, the risk of GV in the second year of the pandemic was similar to the first year [intensity ratio (IR) = 0.996; 95% CI 0.98, 1.01; *p* = 0.53, Figure 1]. GV risk in the US was higher in March through May of 2021 compared to a similar timeframe in 2020 and then declined from June through December 2021. Nine (18%) states registered a significantly higher risk of GV during the second year of the pandemic compared to the same period in the first year (Figure 2). These states were Minnesota, Washington, Georgia, District of Columbia, Alabama, Wisconsin, New York, Texas, and Louisiana. States with a 20% higher risk of gun violence included Minnesota, Washington, and Georgia. In 10 (20%) states, the risk of GV during the second year of the pandemic was significantly lower compared to the same period in the first year: Montana, West Virginia, Missouri, Kansas, Utah, Massachusetts, Arkansas, Michigan, Indiana, and Florida. In the remaining 32 (62%) states, the risk of gun violence in the second year of the pandemic remained the same as of the first year.

**Figure 3:**
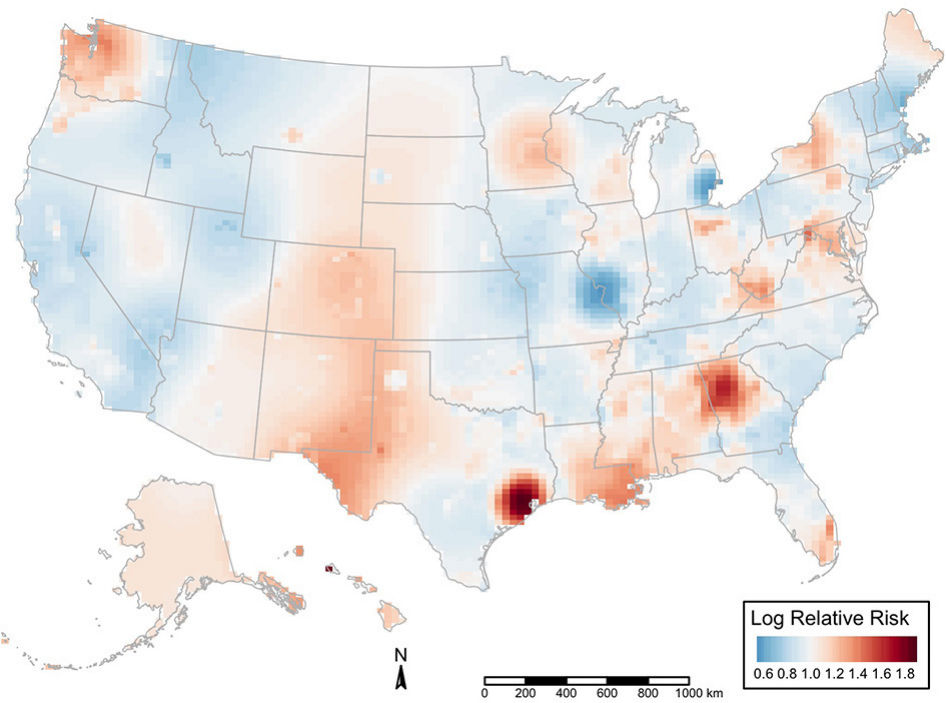
Spatial relative risk of gun violence during the second year of the pandemic vs. the first year. Map shows the intensity (or risk) difference which was estimated by comparing the smoothed intensity of GV events during the second year of the pandemic (March 01, 2021, through February 28, 2022) vs. before the first year of the pandemic (March 1, 2020, through February 28, 2021) across 50 states and the District of Columbia. If the difference is ~0, the risk of GV is unrelated to spatial location. Evidence of spatial variation in risk occurs where the intensities differ. Difference values >0 indicate increased risk, and values < 0 indicate lower risk.

We examined the spatial distribution of GV using the global positioning system coordinates of the events. There were hotspots of higher GV risk within some states (*p* < 0.01). These clusters are heterogeneous (Figure 3).

## Discussion

The results of the present study suggest that GV in the second year of the pandemic increased in 18% of the states, remained the same in 62% of the states, and declined in 20% of the states. States with a 20% higher risk of GV included Minnesota, Washington, and Georgia. The observed increase was most pronounced in the first quarter of 2021.

Putting the results in perspective, over 50% of the states registered an increased rate of gun violence in the first year of the pandemic compared to the pre-pandemic period (1). In the previous study, it was postulated that the stay-at-home orders enacted in March 2020 affected ~96% of the population in the US in the first year of the pandemic and might have bred the ground for increased psychological distress (6), depression (7), increased rates of domestic violence (8), disruptions of social networks (9) and unemployment (10, 11), consequently increased the rates of the gun violence in the first year of the pandemic. However, it is likely that lifting these stay-home mandates in response to the availability of highly effective vaccines could have contributed to the plummeting and reduction of gun violence rates in most of the states (12). The observed increase in GV in some states could be driven by the long-term sequelae of acute SARS-CoV-2 infection, which include mental health disorders (13), although it's known that individuals with mental disorders are more likely to be the victims than perpetrators of GV. The results of the present analysis reinforce the need to promote multiple public health interventions to curb the high rates of GV in the United States. A recent study using a similar dataset found large-scale racial disparities in child exposure to neighborhood firearm violence which worsened during the pandemic (2). Therefore, equitable access to trauma-informed programs is critical to not only mitigate the burden of GV but to facilitate treatment and rehabilitation of the communities that are disproportionately affected.

Gun violence rates declined in 20% of the states. It's plausible actionable public health interventions and effective policies prompted the decline in these regions. Therefore, states that are still grappling with the public health crisis of GV could learn from those with success stories. One major limitation of the present study is the presence of uncertainties in the reported GV events. The gun violence archive is based on news stories and not an official catalog of firearm events. Therefore, biases in this archive may exist due to differences in how journalists cover firearm events. Nevertheless, this is the largest source of data on GV in the US.

## Conclusions

GV risk in the US is heterogeneous and continues to be a public health crisis, with 18% of the states demonstrating significantly higher GV rates during the second year of the COVID-19 pandemic than 1 year prior.

## Data availability statement

Publicly available datasets were analyzed in this study. This data can be found here: https://www.gunviolencearchive.org.

## Author contributions

PS had full access to all the data in the study and takes responsibility for the integrity of the data and the accuracy of the data analysis. PS and VMC: concept and design, critical revision of the manuscript for important intellectual content, and statistical analysis. AS, ESH, PS, and VMC: acquisition, analysis, or interpretation of data. PS drafting of the manuscript and supervision. All authors contributed to the article and approved the submitted version.
